# Relationship between Hyperuricemia and Lipid Profiles in US Adults

**DOI:** 10.1155/2015/127596

**Published:** 2015-01-05

**Authors:** Tao-Chun Peng, Chung-Ching Wang, Tung-Wei Kao, James Yi-Hsin Chan, Ya-Hui Yang, Yaw-Wen Chang, Wei-Liang Chen

**Affiliations:** ^1^Division of Family Medicine, Department of Family and Community Medicine, Tri-Service General Hospital and School of Medicine, National Defense Medical Center, No. 325, Section 2, Chenggong Road, Neihu District, Taipei City 114, Taiwan; ^2^Division of Geriatric Medicine, Department of Family and Community Medicine, Tri-Service General Hospital and School of Medicine, National Defense Medical Center, No. 325, Section 2, Chenggong Road, Neihu District,Taipei City 114, Taiwan; ^3^Graduate Institute of Medical Sciences, National Defense Medical Center, No. 161, Section 6, Minquan E. Road, Neihu District, Taipei City 114, Taiwan; ^4^Department of Occupational Safety and Hygiene, Fooyin University, Kaohsiung, Taiwan

## Abstract

*Background*. Although the link between hyperuricemia and metabolic syndrome had been recognized, the association of the dyslipidemia among individuals with hyperuricemia remains not comprehensively assessed. *Methods*. Using NHANES III study, we examined the relation between serum lipid profiles and different serum uric acid levels, including serum total cholesterol, LDL cholesterol, triglycerides, HDL cholesterol, apolipoprotein-B, lipoprotein (a), apolipoprotein AI, ratio of triglycerides to HDL cholesterol, and ratio of apolipoprotein-B to AI. *Results*. After adjusting for potential confounders, average differences (95% confidence interval) comparing the top to the bottom (reference) serum uric acid were 0.29 (0.19, 0.39) mmol/L for total cholesterol, 0.33 (0.26, 0.41) mmol/L for triglycerides, 0.14 (0.01, 0.27) mmol/L for LDL cholesterol, −0.08 (−0.11, −0.05) mmol/L for HDL, and 0.09 (0.05, 0.12) g/L for serum apolipoprotein-B. Notably, ratios of triglycerides to HDL cholesterol and apolipoprotein-B to AI were also linearly associated with uric acid levels (*P* for trend < 0.001). *Conclusions*. This study suggested that serum LDL cholesterol, triglycerides, total cholesterol, apolipoprotein-B levels, ratio of triglycerides to HDL cholesterol, and ratio of apolipoprotein-B to AI are strongly associated with serum uric acid levels, whereas serum HDL cholesterol levels are significantly inversely associated. In the clinical practice, the more comprehensive strategic management to deal with dyslipidemia and hyperuricemia deserves further investigation.

## 1. Introduction

Serum uric acid is a strong predictor of stroke [1], coronary artery disease [2], and metabolic syndrome [3]. However, the definite role of uric acid in these diseases is still the subject of much discussion and debate because it is always accompanied with other risk factors such as diet, obesity, and dyslipidemia. Specifically, disputation exists about whether serum uric acid is a causative risk factor or only a coexisting marker of those pathologic processes. The paper published by Framingham Heart Study group argued that the relationship between serum uric acid and cardiovascular disease (CVD) is weak, inconsistent with clinical presentations [4]. The relationship between serum uric acid and dyslipidemia is also complex and not fully elucidated. The objective of our study was to investigate the independent relation between serum uric acid and lipid profiles using by The Third National Health and Nutrition Examination Survey (NHANES III), which represents a well-designed population-based study with a large sample size of US adults.

## 2. Methods

### 2.1. Study Population

Executed during the period between 1988 and 1994, the NHANES III consists of a representative sample of the noninstitutionalized civilian US population, which was selected by using a multistage, stratified sampling, and cluster sampling design [5]. All participants were interviewed for demographic, health, and dietary information. After a detailed home-based interview, participants were invited to receive pertinent examination sessions where blood specimens were collected. For participants who were unable to attend the examination for health reasons, a blood sample was obtained during the home interview. We limited our analysis to participants aged 20 years or older who attended the medical examination and included the 14130 eligible subjects (6752 men and 7378 women) with complete information. The NHANES III study received NCHS Institutional Review Board approval, and informed consent was acquired from participants prior to starting the study.

### 2.2. Serum Uric Acid and Lipids Measurements

The level of serum uric acid was measured by using the Hitachi 737 automated multichannel chemistry analyzer (Boehringer Mannheim Diagnostics, Indianapolis, IN, USA). Details concerning data quality control have been published elsewhere [6]. Chemical analyses of total cholesterol, triglycerides, and HDL cholesterol (Hitachi 704 Analyzer) were performed by the Lipoprotein Analytical Laboratory at Johns Hopkins University, Baltimore, Maryland. LDL cholesterol levels were calculated using the Friedewald formula. Both apolipoprotein-B and apolipoprotein AI were measured by radial immunodiffusion (RID) or by Rrate immunonephelometric assay (RIA). All measurements were made with standardized methods having documented accuracy with respect to Centers for Disease Control and Prevention (CDC) reference methods for lipids and lipoproteins.

### 2.3. Assessment of Covariants

The information of daily intakes of cholesterol, total fat, saturated fatty acids, protein, carbohydrate, and total energy intake was based on Dietary Food Frequency Questionnaire [7]. Reliability and validity of Dietary Food Frequency Questionnaire for dietary had been well assessed previously [8, 9]. The participants were interviewed to collect information on age, gender, race, body measurements (including height, weight, and waist), antihyperlipidemic agent, and medical conditions (including self-reported physician-diagnosed diabetes and hypertension). Waist circumference was measured by trained NHANES staff using standard protocols. A brief questionnaire was used to determine the status and amounts of alcoholic beverage. Serum cotinine levels were measured by isotope-dilution high-performance liquid chromatography in tandem with mass spectrometry. Detailed specimen collection and processing instructions are discussed in the NHANES Laboratory Procedures Manual and are available on the NHANES website [6].

### 2.4. Statistical Analysis

All statistical analyses were computed using SPSS Complex Samples (Version 18.0 for Windows, SPSS, Inc., Chicago, IL, USA) to incorporate sample weights and adjust for clusters and strata of the complex sample design. We used quintile-based analysis by dividing serum uric acid levels into quintiles with the subjects in the lowest one as the reference group. The cut-off levels for serum uric acid levels quintiles were as follows: *Q*1 ≦ 238 *μ*mol/L, 238 *μ*mol/L < *Q*2 ≦ 286 *μ*mol/L, 286 *μ*mol/L < *Q*3 ≦ 327 *μ*mol/L, 327 *μ*mol/L < *Q*4 ≦ 381 *μ*mol/L, and 381 *μ*mol/L < *Q*5. We used linear regression modeling to evaluate the relationship between uric acid and lipid levels. We used 3 models with progressive degrees of adjustment. Model 1 was adjusted for age, gender, and race. Model 2 was further adjusted for waist, hypertension, diabetes, drink, cotinine level, and antihyperlipidemic agent use. Model 3 was adjusted for intake of cholesterol, total fat, saturated fatty acids, protein, carbohydrate, and total energy.

## 3. Results

The population's mean age was 44 years. The mean serum uric acid level was 315.84 *μ*mol/L. The characteristics of the study subject quintiles by serum uric acid levels are summarized in Table 1. When serum uric acid levels increased gradually, age; the proportion of men; body mass index; waist; history of hypertension; use of lipid lowering agents; intake of alcohol; cholesterol; total fat; saturated fatty acids; protein; carbohydrate; and total energy tended to increase. Higher serum LDL cholesterol, triglycerides, total cholesterol, apolipoprotein-B levels, and ratio of triglycerides to HDL cholesterol and apolipoprotein-B to AI levels were positively correlated with higher serum uric acid levels, whereas serum HDL cholesterol levels are inversely correlated. After adjusting for age, gender, and race (model 1), serum total cholesterol, triglycerides, LDL cholesterol, and apolipoprotein-B levels in individuals in the highest quintile of serum uric acid levels were higher than in the lowest quintile by 0.46 mmol/L (95% CI 0.41, 0.52; *P* for trend < 0.001), 0.66 mmol/L (95% CI 0.62, 0.71; *P* for trend < 0.001), 0.24 mmol/L (95% CI 0.16, 0.32; *P* for trend < 0.001), and 0.15 g/L (95% CI 0.13, 0.17; *P* for trend < 0.001), respectively. Serum HDL cholesterol in the highest quintile of serum uric acid levels was lower than in the lowest quintile by 0.16 mmol/L (95% CI 0.14, 0.17; *P* for trend < 0.001). The correlation remained unchanged after additionally adjusting for other covariates in models 2 and 3 (Table 2). However, serum lipoprotein (a) and serum apolipoprotein AI are weakly associated with serum uric acid levels after additionally adjusting for other covariates in models 2 and 3.

## 4. Discussion

To the best of our knowledge, there are few studies which focused on the trend of the lipid panels at different levels of uric acid in a nationally representative sample of US adults. Our study illustrated the strong association between serum uric acid and lipid profiles by grading and comprehensively adjusting for confounders. In a survey of 60 patients, Sarmah and Sharma pointed out that serum uric acid levels were associated with the levels of LDL and HDL [10]. A limitation of the previous study was the relatively small sample size, which cannot detect subtle difference of lipid profiles in the clinical setting.

Several important implications can be drawn from our research. First, the level of serum uric acid increased accompanied with increment of serum LDL cholesterol, triglycerides, total cholesterol, and apolipoprotein-B levels. Second, ratios of triglycerides to HDL cholesterol and apolipoprotein-B to AI were also significantly associated with increased uric acid level. Third, there was a strongly inverse relationship between serum uric acid and HDL cholesterol levels regardless of adjustment for sex and several potential confounders, including dietary, hypertension, diabetes, and health related information, suggesting a crucial role of uric acid in the regulation of dyslipidemia. These finding strengthened on previous studies that showed a pathogenesis overlap among hyperuricemia and dyslipidemia [11, 12]. When establishing the diagnosis of hyperuricemia, especially at higher levels, clinical suspicion of coexistent dyslipidemia should be required. These abnormalities had a close relationship to coronary artery disease (CAD) and deserved to be taken seriously.

LDL cholesterol, apolipoprotein-B, and ratio of apolipoprotein-B to AI showed linear correlation with serum uric acid even after adjusting covariants. According to earlier published data, apolipoprotein-B represented a better indicator of the truly LDL particle numbers and CAD [13]. In the prospective studies conducted by Walldius and McQueen, the ratio of apolipoprotein-B to AI was also demonstrated to be one of the strongest risk predictors for cardiovascular events [14, 15]. These findings strengthen the evidence about the relationship among serum uric acid, dyslipidemia, and CAD risk. It is controversial if serum uric acid is only a marker of preexisting disorder or a causal factor for dyslipidemia and CAD. Increased apolipoprotein-B to AI ratio and insulin levels were evidenced to lower eGFR or decrease renal excretion of uric acid [16, 17]. Therefore, these would lead to decreasing uric acid excretion by urine, which cause further hyperuricemia. However, hyperuricemia can affect adipocytes by increasing monocyte chemoattractant protein 1 and reducing production of adiponectin, thereby contributing to insulin resistance and inflammation [18–20]. In our study, the triglycerides to HDL cholesterol ratio, a reliable indicator of insulin resistance, also showed the positive correlation with serum uric acid. Similarly, previous study revealed in full detail that serum uric acid was associated with increased triglycerides to HDL cholesterol ratio [21]. Decreased insulin resistance in leptin related obesity and fructose-induced metabolic syndrome was noted when lowering uric acid by uricosuric agents and xanthine oxidase inhibitors [18, 22, 23]. The above-mentioned findings highlighted complex interaction between serum uric acid and lipids.

In our study, serum HDL cholesterol, as a protective factor for CVD risk, is inversely related to uric acid level in line with previous study. It has been lately noted that elevated serum uric acid was a significant predictor of smaller, denser LDL cholesterol and HDL cholesterol particles, which offers a greater atherogenic ability [24]. The decline of HDL cholesterol will give rise to the formation of atherosclerosis and eventually predisposed to cardiovascular disease (CVD), but the direct evidence that increasing HDL cholesterol is beneficial in reducing cardiovascular events has not been established. On the other hand, the connections between triglycerides and uric acid levels were linear and evident [12, 25]. This was in complete agreement with our findings. The relationship between triglycerides and uric acid level had been attributed to genetic factors [26, 27]. It is tempting to speculate that the synthesis of triglycerides will need NADPH, which resulted in increased uric acid production [28].

It is now widely acceptable to receive antihyperlipidemic drugs to lower the CVD risk. The latest therapeutic strategies for hyperlipidemia attended to risk reduction, instead of the target of exactly lipid level such as LDL cholesterol level for 70 mg/dL [29]. Our study showed strong relationship between uric acid and these lipid profiles. It seems that we cannot just take lipid into account and let uric acid behind. Collectively, our study implied that uric acid might intensify many pathophysiological mechanisms associated with the risk CVD and might have synergistic interactions with other lipid profiles causing CVD. Due to the strong concurrence of dyslipidemia and hyperuricemia, it is urgent to develop appropriate treatment guidelines such as life style modification, diet, and pharmacologic measures taking into account improving hyperuricemia and holistic long-term health effects. Furthermore, in more recent years, prevalence of hyperuricemia was predisposed by the increasing frequency of risk factors, such as obesity and metabolic syndrome [30]. These abnormalities influenced each other by diverse mechanism and precipitated by similar factors such as diet, life style, and genes. Detection and treatment of disordered lipid and uric metabolism in patients with multiple risk factors for CVD should be given a high priority in the clinical setting.

The current analysis had few limitations. This study was performed in a nationally representative sample of US general population; therefore, the findings are likely to be generalizable to the US general population. Although previous reports and biological plausibility consistently suggest that lipid levels would be associated with the serum uric acid levels as observed, a cross-sectional study design tends to leave uncertainty regarding the temporal sequence of exposure, outcome relations. Thus, confirming the relation with prospective longitudinal data (e.g., relation between prior dyslipidemia and incident hyperuricemia) would be valuable. Further investigation of the potentially modifiable impact of apolipoprotein-B or lipoprotein (a) would also be warranted, including clinical trials.

## 5. Conclusion

From a nationally representative sample of US adults, our study demonstrated that serum LDL cholesterol, triglycerides, total cholesterol, apolipoprotein-B levels, ratio of triglycerides to HDL cholesterol, and ratio of apolipoprotein-B to AI are significantly associated with serum uric acid levels, whereas serum HDL cholesterol levels are inversely associated. The more comprehensive strategic management to deal with dyslipidemia and hyperuricemia deserves further investigation.

## Figures and Tables

**Table 1:** Characteristics of the study population by serum uric acid quintiles.

	Serum uric acid levels (*μ*mol/L)						
	Overall	1st (≦238)	2nd (239–286)	3rd (287–327)	4th (328–381)	5th (≧382)	*P* for trend
*N*	14130	2749	2764	2674	2843	3100	
Uric acid (*μ*mol/L)	315.84	205.11	265.21	308.72	355.69	442.53	<0.001
Age (years)	44.08	40.87	43.01	44.41	44.95	47.12	<0.001
Sex (% male)	49.10	10.40	30.30	53.80	70.50	79.00	<0.001
Race (% white)	84.90	85.50	84.40	85.60	85.80	83.20	0.21
Body mass index (Kg/m^2^)	26.63	24.01	25.45	26.97	27.55	29.11	<0.001
Waist (cm)	92.07	82.69	88.20	92.95	95.84	100.59	<0.001
History of hypertension (%)	23.00	12.70	20.10	22.80	23.30	36.00	<0.001
History of diabetes (%)	5.00	6.20	4.70	5.00	3.70	6.20	0.49
Drink/month	29.00	15.10	24.50	26.90	34.20	39.30	<0.001
Serum cotinine (nmol/L)	451.84	430.77	458.49	476.21	486.83	407.94	0.79
Antihyperlipidemic drug use (%)	2.20	1.40	1.40	1.60	2.60	3.80	<0.001
Average daily intakes							
Calories (kcal/d)	2223.89	1909.78	2082.67	2340.35	2410.76	2378.32	<0.001
Protein (g/d)	83.28	70.46	76.84	87.74	90.32	91.09	<0.001
Total carbohydrates (g/d)	271.20	240.26	259.35	283.73	288.76	284.33	<0.001
Total fats (g/d)	85.37	74.00	80.09	91.46	92.68	88.89	<0.001
Cholesterol (mg/d)	288.26	254.80	265.24	301.18	312.09	307.88	<0.001
Total saturated fatty acids (g/d)	28.58	25.22	26.47	31.10	30.88	29.36	<0.001
Serum lipids and apolipoproteins							
Total cholesterol (mmol/L)	5.21	4.93	5.16	5.24	5.33	5.39	<0.001
Total cholesterol (% of risk)^*^	48.4	38	44.7	49.8	52.7	55.6	
Triglycerides (mmol/L)	1.49	1.15	1.35	1.44	1.63	1.84	<0.001
Triglycerides (% of risk)^*^	16.6	7.9	12	14.5	18.5	27.4	
HDL cholesterol (mmol/L)	1.25	1.38	1.32	1.25	1.18	1.13	<0.001
HDL cholesterol (% of risk)^*^	26.3	13.6	19.8	25.4	31.2	39.4	
LDL cholesterol (mmol/L)	3.32	3.08	3.22	3.35	3.46	3.45	<0.001
LDL cholesterol (% of risk)^*^	76.6	68.5	74.1	78.9	81.6	80.1	
Apolipoprotein-B (g/L)	1.05	0.96	1.01	1.05	1.09	1.14	<0.001
Apolipoprotein-B (% of risk)^*^	23.6	14.1	19.8	22.5	27.1	34.5	
Lipoprotein (a) (g/L)	0.23	0.22	0.23	0.24	0.22	0.22	0.424
Lipoprotein (a) (% of risk)^*^	32.6	30.3	32.5	33.2	32	34.7	
Apolipoprotein AI (g/L)	1.40	1.46	1.44	1.39	1.36	1.36	<0.001
Apolipoprotein AI (% of risk)^*^	0.6	0.7	0.5	0.6	0.4	0.8	

Data are survey-weighted means or percentages for continuous or categorical variables, respectively.

^*^Risk values of serum lipids and apolipoproteins: total cholesterol >5.18 mmol/L, triglycerides >2.26 mmol/L, HDL cholesterol <1.03 mmol/L, LDL cholesterol >2.59 mmol/L, serum apolipoprotein-B >1.25 g/L, serum lipoprotein (a) >0.3 g/L, and serum apolipoprotein AI <0.94 g/L.

**Table 2:** Adjusted differences (95% CI) in serum lipids, triglycerides to HDL cholesterol ratio, and apolipoprotein-B to AI ratio comparing the four higher quartiles to the first quartile of serum uric acid.

	Serum uric acid levels (*μ*mol/L)					
	1st (≦238)	2nd (239–286)	3rd (287–327)	4th (328–381)	5th (≧382)	*P* for trend
Total cholesterol (mmol/L)						
Model 1	0.00 (Reference)	0.22 (0.17, 0.27)	0.32 (0.26, 0.37)	0.43 (0.37, 0.49)	0.46 (0.41, 0.52)	<0.001
Model 2	0.00 (Reference)	0.18 (0.10, 0.27)	0.19 (0.10, 0.28)	0.24 (0.15, 0.33)	0.30 (0.20, 0.39)	<0.001
Model 3	0.00 (Reference)	0.17 (0.09, 0.26)	0.19 (0.10, 0.28)	0.23 (0.14, 0.32)	0.29 (0.19, 0.39)	<0.001
Triglycerides (mmol/L)						
Model 1	0.00 (Reference)	0.18 (0.14, 0.22)	0.28 (0.23, 0.32)	0.46 (0.41, 0.50)	0.66 (0.62, 0.71)	<0.001
Model 2	0.00 (Reference)	0.12 (0.06, 0.18)	0.11 (0.05, 0.18)	0.25 (0.19, 0.32)	0.33 (0.26, 0.40)	<0.001
Model 3	0.00 (Reference)	0.12 (0.06, 0.18)	0.10 (0.03, 0.17)	0.27 (0.20, 0.33)	0.33 (0.26, 0.41)	<0.001
HDL cholesterol (mmol/L)						
Model 1	0.00 (Reference)	−0.04 (−0.05, −0.02)	−0.08 (−0.09, −0.06)	−0.12 (−0.13, −0.10)	−0.16 (−0.17, −0.14)	<0.001
Model 2	0.00 (Reference)	−0.02 (−0.04, 0.00)	−0.05 (−0.08, −0.03)	−0.07 (−0.10, −0.05)	−0.08 (−0.11, −0.05)	<0.001
Model 3	0.00 (Reference)	−0.01 (−0.04, 0.01)	−0.05 (−0.07, −0.02)	−0.08 (−0.10, −0.05)	−0.08 (−0.11, −0.05)	<0.001
LDL cholesterol (mmol/L)						
Model 1	0.00 (Reference)	0.10 (0.03, 0.18)	0.19 (0.12, 0.27)	0.28 (0.21, 0.36)	0.24 (0.16, 0.32)	<0.001
Model 2	0.00 (Reference)	0.08 (−0.04, 0.19)	0.13 (0.01, 0.25)	0.21 (0.09, 0.33)	0.14 (0.01, 0.27)	0.015
Model 3	0.00 (Reference)	0.08 (−0.03, 0.20)	0.13 (0.01, 0.24)	0.20 (0.08, 0.33)	0.14 (0.01, 0.27)	0.022
Apolipoprotein-B (g/L)						
Model 1	0.00 (Reference)	0.05 (0.03, 0.07)	0.08 (0.06, 0.10)	0.12 (0.10, 0.14)	0.15 (0.13, 0.17)	<0.001
Model 2	0.00 (Reference)	0.04 (0.01, 0.07)	0.03 (0.00, 0.06)	0.06 (0.03, 0.09)	0.08 (0.05, 0.12)	<0.001
Model 3	0.00 (Reference)	0.04 (0.02, 0.07)	0.03 (0.00, 0.06)	0.06 (0.03, 0.09)	0.09 (0.05, 0.12)	<0.001
Lipoprotein (a) (g/L)						
Model 1	0.00 (Reference)	0.01 (−0.12, 0.03)	0.02 (0.00, 0.04)	0.01 (−0.01, 0.03)	0.00 (−0.02, 0.02)	0.962
Model 2	0.00 (Reference)	0.01 (−0.02, 0.04)	0.02 (−0.02, 0.05)	0.02 (−0.01, 0.05)	0.02 (−0.02, 0.05)	0.373
Model 3	0.00 (Reference)	0.01 (−0.03, 0.04)	0.01 (−0.03, 0.04)	0.01 (−0.02, 0.05)	0.00 (−0.03, 0.04)	0.559
Apolipoprotein AI (g/L)						
Model 1	0.00 (Reference)	−0.01 (−0.02, 0.01)	−0.03 (−0.05, −0.01)	−0.04 (−0.06, −0.02)	−0.04 (−0.06, −0.02)	<0.001
Model 2	0.00 (Reference)	0.03 (0.01, 0.05)	−0.03 (−0.05, 0.00)	−0.01 (−0.03, 0.02)	0.02 (−0.01, 0.05)	0.740
Model 3	0.00 (Reference)	0.03 (0.00, 0.05)	−0.02 (−0.05, 0.00)	−0.01 (−0.04, 0.02)	0.01 (−0.02, 0.04)	0.738
Triglycerides to HDL cholesterol ratio						
Model 1	0.00 (Reference)	0.16 (0.11, 0.21)	0.27 (0.21, 0.32)	0.48 (0.42, 0.53)	0.72 (0.67, 0.78)	<0.001
Model 2	0.00 (Reference)	0.09 (0.01, 0.17)	0.10 (0.02, 0.18)	0.27 (0.19, 0.36)	0.33 (0.25, 0.42)	<0.001
Model 3	0.00 (Reference)	0.08 (0.00, 0.17)	0.08 (0.00, 0.17)	0.29 (0.20, 0.37)	0.35 (0.0.26, 0.44)	<0.001
Apolipoprotein-B to AI ratio						
Model 1	0.00 (Reference)	0.04 (0.02, 0.05)	0.07 (0.06, 0.09)	0.11 (0.09, 0.12)	0.14 (0.12, 0.15)	<0.001
Model 2	0.00 (Reference)	0.02 (0.00, 0.04)	0.04 (0.01, 0.06)	0.05 (0.02, 0.08)	0.06 (0.03, 0.09)	<0.001
Model 3	0.00 (Reference)	0.02 (0.00, 0.05)	0.03 (0.01, 0.06)	0.05 (0.03.0.08)	0.06 (0.03, 0.09)	<0.001

Adjusted covariates: model 1 = age, gender, and race; model 2 = model 1 + waist, hypertension, diabetes, alcohol drink, cotinine level, and antihyperlipidemic drug use; model 3 = model 2 + intake of total energy (kcal/day), protein (g/day), carbohydrates (g/day), total fats (g/day), cholesterol (mg/day), and total saturated fatty acids (g/day).
